# Effects of a Fruit- and Vegetable-Enriched Breakfast on Cognition, Attention, and Mood in Primary School Children: A Randomized Controlled Trial

**DOI:** 10.3390/nu18040581

**Published:** 2026-02-10

**Authors:** Wenyun Li, Xiaotian Du, Yuanwei Ma, Huliang Cao, Shan Jin, Jing Fan, Jian Gao, Min Hou, Bo Chen

**Affiliations:** 1 Department of Clinical Nutrition, Zhongshan Hospital, Fudan University, Shanghai 200032, China; li.wenyun@zs-hospital.sh.cn (W.L.); gao.jian@zs-hospital.sh.cn (J.G.); 2 Department of Nutrition & Food Hygiene, School of Public Health, Key Laboratory of Public Health Safety of the Ministry of Education, Fudan University, Shanghai 200032, China; 24111020084@m.fudan.edu.cn (X.D.); 23211020138@m.fudan.edu.cn (S.J.); 21111020063@m.fudan.edu.cn (J.F.); 3 School of Public Health, College of Medicine, Shanghai Jiao Tong University, Shanghai 200025, China; yuanwei_ma@163.com; 4 Shanghai Food Industry Institute, Shanghai 200090, China; caohuliang@126.com

**Keywords:** children, breakfast, fruits and vegetables, cognition, randomized controlled trial

## Abstract

**Background/Objectives**: Increasing fruit and vegetable (F&V) intake may benefit children’s neurobehavioral development, but randomized evidence remains limited. We evaluated whether F&V-enriched breakfast improves cognition, attention and mood in primary school children. **Methods**: We conducted a 12-week class-randomized controlled trial in Shanghai, China. A total of 251 children aged 7–11 years received either a daily F&V-enriched breakfast or a standardized control breakfast with comparable energy provision, both centrally prepared and home-delivered. The primary outcome was change in Full-Scale IQ (FSIQ) measured by the Wechsler Intelligence Scale for Children—Fourth Edition (WISC-IV). Secondary outcomes included WISC-IV composite indices, attention-related behavioral symptoms assessed by the Parent Symptom Questionnaire, and mood by the Profile of Mood States—Brief. Analyses followed the intention-to-treat principle with multiple imputation. **Results**: Among 282 children screened, 251 were randomized, with 243 completing post-intervention assessments and 230 completing follow-up. The adjusted mean change in FSIQ did not differ between groups (mean difference = −0.63; 95% CI −5.15~3.89; *p* = 0.807). No statistically significant between-group differences in change were observed for other cognitive indices, behavioral symptoms, or overall mood disturbance. Exploratory analyses suggested a greater reduction in fatigue scores among children aged ≤8 years in the intervention group compared with controls. Dietary assessment confirmed higher dietary fiber and carotene intakes in the intervention group at week 12. No intervention-related adverse events were reported. **Conclusions**: An F&V-enriched breakfast improved dietary quality but did not produce measurable between-group improvements in cognitive or neurobehavioral outcomes over 12 weeks. Exploratory age-specific findings warrant further investigation.

## 1. Introduction

Childhood is a critical period for neurobehavioral development, characterized by rapid changes in cognition, emotional regulation, and attentional control. A growing body of evidence suggests that modifiable lifestyle factors, particularly diet, play a crucial role in shaping these developmental trajectories. Among dietary components, fruits and vegetables (F&V) have attracted increasing attention due to their richness in vitamins, minerals, dietary fiber, and bioactive compounds that support brain function and protect against oxidative stress. Despite national and international dietary guidelines underscoring their importance, many children fail to consume adequate amounts. Globally, fewer than 40% of adolescents report eating fruits or vegetables daily, and more than half do not meet even minimal daily recommendations [1]. In China, the 2022 Dietary Guidelines recommend approximately 300 g of vegetables and 150–200 g of fruit per day for school-aged children, yet actual intake is substantially lower [2]. A recent survey in Shanghai found that nearly half of students did not consume fruit daily, and about one quarter did not consume vegetables daily [3]. This gap between recommendations and practice has raised concerns that insufficient F&V consumption may adversely affect children’s health and well-being.

Observational studies consistently link higher F&V intake with improved neurocognitive outcomes in children. Large-scale investigations in Western countries report that dietary patterns rich in F&V are associated with enhanced memory, attention, and academic performance [4]. In the United Kingdom, a study of nearly 9000 pupils found that achieving a “five-a-day” intake was positively associated with mental well-being and cognitive functioning at school [5]. Similar associations have been observed in Australia and Europe, where higher F&V consumption correlates with improved test scores and executive function [6]. Evidence from China is consistent. Analyses of children and adolescents indicate that nutrient-dense, produce-rich diets are associated with superior academic performance, whereas Westernized, high-fat dietary patterns are linked to poorer outcomes [4].

Beyond global cognition and academic achievement, diet quality has been tied to attention and behavioral regulation. Among children with attention-deficit/hyperactivity disorder (ADHD) symptoms, higher F&V intake has been associated with less severe inattention, while diets high in saturated fats and added sugars have been linked to worse symptom profiles [7]. A recent meta-analysis further confirmed that healthy dietary patterns rich in F&V are protective against ADHD, whereas unhealthy patterns high in refined sugars and fats increase risk [8]. Among typically developing children, greater F&V intake has been linked to better attention spans, fewer behavioral problems, and enhanced emotional regulation in classroom settings [9]. Diet also appears to influence mood and emotional well-being. Multiple studies have reported that higher F&V intake is associated with fewer depressive and anxiety symptoms, greater life satisfaction, and more positive affect [10], while diets high in ultra-processed snacks and sugar-sweetened beverages are associated with increased emotional difficulties and a higher risk of mood disturbances [11].

Breakfast habits represent an additional, independent factor influencing cognitive and emotional outcomes. Regular breakfast consumption has been linked to better academic performance, improved memory and attention, and more stable moods in school-aged children [12,13]. Conversely, skipping breakfast has been associated with impaired attention, heightened irritability, and increased psychosocial distress [14]. These findings have motivated the implementation of school breakfast programs worldwide. While such programs can alleviate morning hunger and improve attendance and classroom behavior, they frequently face challenges related to food preparation infrastructure, participation rates, and menu quality, including limited access to fresh produce. Strengthening breakfast quality by routinely incorporating F&V may amplify the benefits attributed to both breakfast consumption and F&V intake.

Despite strong rationale and consistent observational evidence, key knowledge gaps remain. Most studies examining the relationship between F&V intake and neurobehavioral outcomes in children are observational or conducted in high-income settings. Few intervention trials have tested whether increasing F&V consumption can causally improve cognition, attention, or mood in school-aged children, particularly in low- and middle-income contexts. Within China, empirical data remain limited, even as national surveys consistently document inadequate F&V intake among children. Implementation strategies that are culturally and logistically feasible are needed to overcome barriers such as taste preferences, food safety, and limited breakfast infrastructure in schools. Furthermore, although previous interventions have often relied on supplementation or addition strategies, simply adding fruits or vegetables on top of existing meals, such approaches alter total food quantity without addressing real-world dietary substitution.

To bridge this gap, we adopted a substitution-based design, replacing customary breakfast side dishes with F&V while maintaining overall nutritional adequacy. The trial was pragmatic and school-recruited but implemented through daily home-delivered breakfasts, ensuring consistent quality and adherence. We tested whether this 12-week replacement improved children’s dietary quality and led to measurable changes in cognition, attention, and mood, with persistence evaluated at short-term follow-up. The primary outcome was change in the Chinese Wechsler Intelligence Scale for Children—Fourth Edition (WISC-IV) Full-Scale IQ (FSIQ) from baseline to week 12; secondary outcomes were attention and mood. By evaluating a culturally tailored and operationally scalable substitution model, this study provides trial evidence on feasibility and dietary impact that can support the development and evaluation of breakfast nutrition strategies and school feeding programs in China.

## 2. Materials and Methods

### 2.1. Study Design and Participants

This 12-week, parallel-group, school-based randomized controlled trial was conducted in Putuo District, a central urban area of Shanghai, China, between 11 September 2024 and 28 March 2025. A total of 251 participants were recruited from one school serving families of moderate socioeconomic status. Eligible participants were full-time students aged 7–11 years who were generally healthy. Exclusion criteria included diagnosed cognitive or developmental disorders, chronic illnesses likely to interfere with participation, or food allergies/dietary restrictions precluding consumption of study-provided meals. Recruitment was conducted in collaboration with the school through classroom briefings, parent information sessions, and distribution of consent forms.

### 2.2. Ethics Statement

The study was approved by the Institutional Review Board of the School of Public Health, Fudan University (IRB00002408; FWA00002399), and prospectively registered at ClinicalTrials.gov (NCT06560359). The study adhered to the principles of the Declaration of Helsinki. Written informed consent was obtained from parents or legal guardians, and all children provided assent.

### 2.3. Randomization and Masking

**Figure 1:**
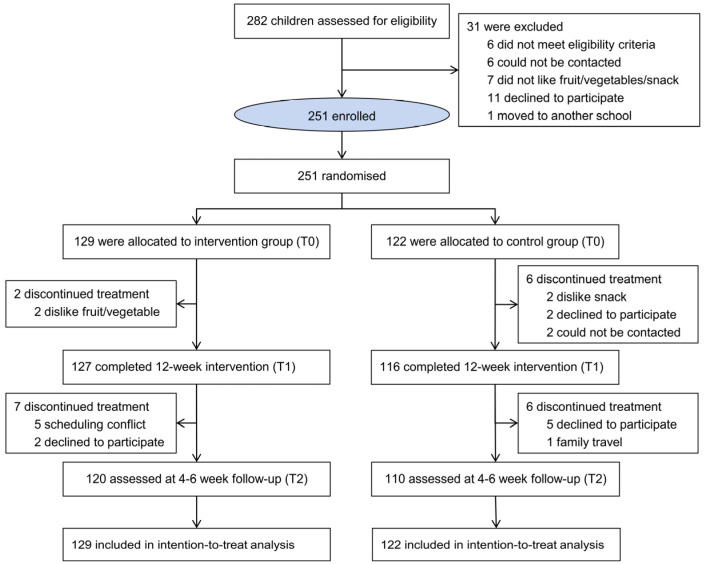
Trial design and assessment schedule of the F&V-enriched breakfast randomized trial. Note: Children were randomized to intervention or control. The intervention replaced the breakfast’s customary side dish with one serving of fruit and one serving of vegetables for 12 weeks. Assessments were obtained at baseline (T0), post-intervention (T1, 12 weeks), and short-term follow-up (T2, 4–6 weeks after the intervention). F&V, fruit and vegetable.

Participants were randomized to the intervention or control arm using a computer-generated sequence with blocking and stratification by grade. Given the nature of the intervention, participants and kitchen/logistics staff were not blinded; however, outcome assessors and data analysts remained masked until completion of the primary analysis. No interim analyses or formal trial-level stopping guidelines were planned. Participants could be withdrawn if adverse reactions such as allergy or gastrointestinal discomfort occurred, or if parents/legal guardians requested discontinuation (Figure 1).

### 2.4. Procedures

All study breakfasts were centrally prepared and delivered daily throughout the 12-week intervention period, including weekends and holidays. Depending on parental preference, meals were either distributed at school or delivered to students’ homes. Trained staff documented delivery and receipt daily, with unannounced spot checks to ensure menu fidelity. Plate waste was quantified in a random subsample using calibrated digital scales to estimate actual intake.

Assessments were conducted at baseline (T0), post-intervention at week 12 (T1), and 4–6 weeks after completion (T2). Data collection followed standardized procedures covering cognitive, attention-related behavioral, emotional, anthropometric, and dietary domains. Cognitive performance was assessed using the Chinese Wechsler Intelligence Scale for Children—Fourth Edition (WISC-IV), adapted for use in mainland China by King-May Psychological Assessment Technology Development Limited Company (Zhuhai, China). The instrument includes four primary cognitive indices, Verbal Comprehension, Perceptual Reasoning, Working Memory, and Processing Speed, which together generate the FSIQ. Two composite indices were also derived. The General Ability Index reflects abilities in Verbal Comprehension and Perceptual Reasoning, and the Cognitive Proficiency Index reflects abilities in Working Memory and Processing Speed. Attention-related behaviors were assessed using the Chinese Parent Conners Symptom Questionnaire (PSQ). A total score was generated, along with five symptom domains: Inattention, Hyperactivity and Impulsivity, Behavioral and Social Problems, Emotional Problems and Somatic Symptoms. Mood was measured using the Chinese Profile of Mood States—Brief (POMS). The Total Mood Disturbance score was calculated from seven mood dimensions: Tension, Anger, Fatigue, Depression, Vigor, Confusion and Esteem-Related Affect.

Anthropometric indicators, including height, weight, and body mass index (BMI)-for-age z-scores, were measured according to WHO standards. Dietary intake was assessed at each time point using three non-consecutive 24-h dietary records, including two weekdays and one weekend day. Parents or guardians were provided with standardized paper forms and calibrated digital kitchen scales, and were instructed to weigh and record all foods and beverages consumed by the child over the entire day. For each item, parents recorded the weight and indicated the eating occasion, including breakfast, lunch, dinner, and snacks. Nutrient intakes were calculated using the China Food Composition Tables, Standard Edition, 6th edition. Interviewers underwent centralized training, and data quality was ensured through inter-rater reliability checks, double data entry, and automated range verification (Supplementary Methods).

The planned sample size provided 80% power (two-sided α = 0.05) to detect a between-group difference of 2.17 points in change in WISC-IV FSIQ, assuming a pooled SD of 4.11, informed by effect sizes reported in a pediatric cognitive intervention trial [15]. This required 75 participants per arm (150 total). Allowing for 20% attrition, the minimum sample size was set at 188. Detailed assumptions and calculations are presented in Supplementary Methods. In practice, recruitment was conducted at the grade level: limiting participation to selected classes would have raised equity concerns among families and complicated logistics. Therefore, 251 children were enrolled, which further increased precision and allowed adequately powered analyses of prespecified secondary and exploratory outcomes.

Adverse events were systematically monitored through daily parental reports and weekly staff checks. No serious adverse events related to the intervention were observed.

### 2.5. Intervention

Two standardized breakfast menus were designed by nutritionists to align with national dietary guidelines for school-aged children, with breakfasts providing approximately one-third of daily energy requirements. At the menu-design stage, the intervention and control breakfasts were planned to be comparable in overall energy provision, using a substitution-based approach. Both groups received a staple item, a Chinese-style side dish, an egg item, and milk. The only difference was the side component: in the control arm, breakfasts included a packaged snack (e.g., nuts, sweet potato, or chips), whereas in the intervention arm, the snack was replaced by one portion of fresh fruit (≈80 g) and one portion of fresh or lightly cooked vegetables (≈60 g). All recipes, specifications, and portion sizes were standardized and delivered in a rotating 30-day cycle. Menus were adjusted seasonally and refined based on parental feedback to ensure cultural acceptability and freshness. Detailed menu rotation, portion standards, and nutrient composition are presented in Supplementary Methods and Tables S1–S3.

### 2.6. Outcomes and Assessments

The primary outcome was change in FSIQ from baseline to week 12, measured by the WISC-IV. Secondary outcomes included attention and behavioral regulation, assessed by the PSQ, and mood, assessed by the POMS. Potential harms such as gastrointestinal discomfort and allergic reactions were recorded through structured parental and staff reports.

### 2.7. Statistical Analysis

Baseline characteristics were summarized using mean and standard deviation (SD) for continuous variables and percentages for categorical variables. All main analyses followed the intention-to-treat (ITT) principle, including all randomized participants according to their assigned group. Primary analyses evaluated the effects of the intervention on cognitive, attention-related behavioral, and mood outcomes using linear mixed-effects models with a random intercept for participant and fixed effects for group, time, and their interaction. Models were adjusted for baseline age, sex, BMI, and household income. Adjusted mean differences and 95% confidence intervals (CI) were estimated for each outcome. To address potential bias from missing data, multiple imputation using chained equations was implemented under the ITT framework. Temporal trajectories were further examined by comparing pre-post and pre-follow-up timepoints using the same model structure.

Sensitivity analyses were conducted to evaluate the robustness of findings. To explore whether intervention effects differed across participant characteristics, subgroup analyses were conducted by sex, age, BMI status, and household income. Subgroup-specific estimates and 95% CIs were summarized using forest plots. Adherence was monitored during the 12-week intervention through parental reports of daily breakfast consumption. In the intervention group, participants were considered non-adherent if both F&V portions were frequently left uneaten (i.e., >50% unfinished on most days) or if either item was consistently untouched throughout the study. In the control group, participants were classified as non-adherent if >75% of the provided breakfast was routinely uneaten. All others were considered adherent. Inverse-probability weighting (IPW) models were applied among adherent participants to correct for potential selection bias, and per-protocol (PP) analyses were conducted among adherent participants with complete covariate data. Descriptive paired and independent *t*-tests were used to summarize unadjusted within- and between-group changes. All tests were two-sided, and statistical significance was set at *p* < 0.05. Analyses were performed using R software (version 4.3.2).

## 3. Results

### 3.1. Participant Flow and Baseline Characteristics

A total of 282 children were assessed for eligibility, and 251 were randomized to the intervention group (*n* = 129) or the control group (*n* = 122) (Figure 1). Post-intervention assessments were completed by 243 participants (96.8%), and 230 participants completed the 4–6-week follow-up. Eight participants withdrew, including two in the intervention group and six in the control group, mainly due to dissatisfaction with meal taste or parental decision to discontinue participation. Baseline demographic characteristics, anthropometric measures, socioeconomic indicators, breakfast habits, and baseline cognitive, attention-related behavioral, and mood scores were well balanced across groups (Table 1 and Table S5A,B).

### 3.2. Primary Cognitive Outcome

Under the ITT framework with multiple imputation, the primary outcome, change in WISC-IV FSIQ from baseline to week 12, did not differ between groups (adjusted mean difference = −0.63 points, 95% CI −5.15~3.89; *p* = 0.807, Table 2).

### 3.3. Secondary Cognitive Outcomes and Subgroup Analyses

**Figure 2:**
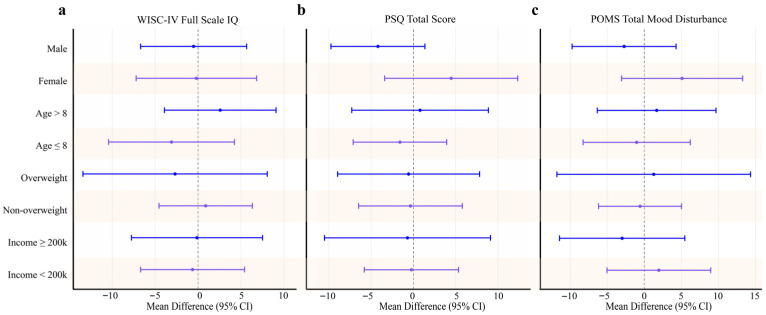
Subgroup analyses of changes in cognitive, behavioral, and mood outcomes. Forest plots display adjusted mean differences (intervention minus control) and 95% confidence intervals for (**a**) WISC-IV Full-Scale IQ, (**b**) PSQ total score, and (**c**) POMS Total Mood Disturbance across predefined subgroups. All estimates were obtained under the ITT framework using multiple imputation to handle missing data. Positive values indicate greater improvement in the intervention group. WISC-IV, Chinese Wechsler Intelligence Scale for Children—Fourth Edition; POMS, Profile of Mood States—Brief; PSQ, Parent Conners Symptom Questionnaire; ITT, intention-to-treat.

As shown in Table 3 and Table S6, the Cognitive Proficiency Index increased in both the intervention (101.93 [15.67] to 112.32 [15.01]; *p* = 0.049) and control groups (104.08 [16.02] to 114.95 [16.36]; *p* = 0.003). However, between-group differences in change were likewise small and non-significant across all WISC-IV indices and composite scores (Table S7). Subgroup analyses by age, sex, BMI status, and household income yielded consistent results (Figure 2).

### 3.4. Behavioral Outcomes

For behavioral outcomes assessed by the PSQ, no significant between-group difference was observed in the change in total PSQ score from baseline to week 12 (adjusted mean difference = −0.18 points, 95% CI −4.99~4.63; *p* = 0.968, Table 2), and between-group differences across individual symptom domains were not statistically significant (Table S7). However, within-group analyses under the ITT framework indicated improvements in selected behavioral domains in the intervention group over the intervention period. In particular, somatic symptoms decreased from baseline to post-intervention in the intervention group (1.53 [1.89] to 1.10 [1.58], *p* = 0.047, Table 3 and Table S6), whereas no corresponding change was observed in the control group. Other PSQ domains showed small and inconsistent within-group changes in both groups.

### 3.5. Mood Outcomes

Mood outcomes assessed by the POMS did not differ significantly between groups. Changes in Total Mood Disturbance from baseline to week 12 were similar in the intervention and control groups (MD = 0.60; 95% CI −4.86~6.06; *p* = 0.927; Table 2), and no consistent between-group differences were observed across individual POMS subscales (Table S7).

### 3.6. Sensitivity and Follow-Up Analyses

Sensitivity analyses using linear mixed-effects models yielded results consistent with the primary ITT analyses (Table S8). At the 4–6-week follow-up, modest improvements relative to baseline were observed in both groups, but no statistically significant between-group differences in change were detected for any outcome (Table S9).

**Figure 3:**
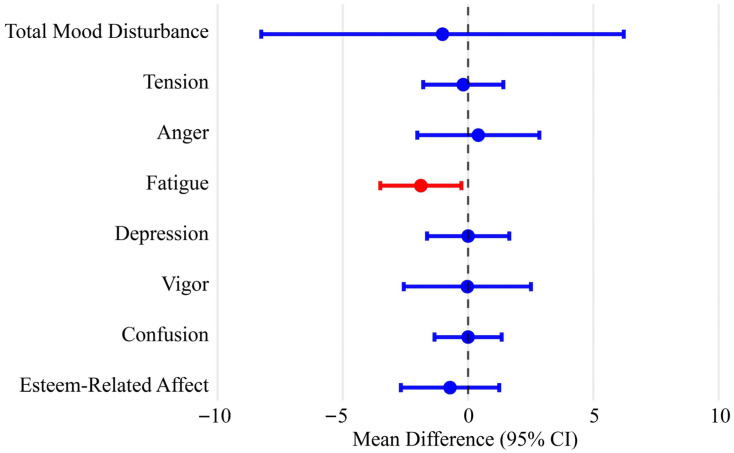
POMS subscale changes in children aged ≤8 years. Forest plot displays adjusted mean differences between intervention and control groups, with 95% confidence intervals, across POMS subscales among children aged ≤8 years. Analyses followed the ITT framework using multiple imputation with covariate adjustment. Negative values indicate greater reductions in symptom scores in the intervention group. POMS, Profile of Mood States—Brief; ITT, intention-to-treat.

In an exploratory age-stratified analysis, children aged 8 years or younger in the intervention group exhibited a greater reduction in POMS Fatigue scores compared with controls (MD = −1.89; 95% CI −3.51~−0.27; *p* = 0.022; Figure 3).

### 3.7. Adherence and Additional Analyses

A total of 228 participants (90.8%) met the adherence criteria and were included in the prespecified sensitivity analyses. Results from IPW and PP analyses were broadly consistent with the main ITT and did not materially alter the overall interpretation (Tables S10 and S11).

### 3.8. Dietary Intake and Intervention Fidelity

Dietary assessment indicated that total energy and macronutrient intakes were similar between groups at baseline and post-intervention (Table S12). At week 12, dietary fiber (15.80 [6.53] vs. 13.68 [5.82] g/day; *p* = 0.027) and carotene (2.04 [1.48] vs. 1.58 [1.06] mg/day; *p* = 0.023) intakes were significantly higher in the intervention group compared with the control group (Table 4), confirming successful implementation of the F&V-enriched breakfast. No adverse events attributable to the intervention were reported.

## 4. Discussion

To our knowledge, this is the first randomized controlled trial to evaluate whether enriching children’s breakfasts with F&V can improve cognition, attention, and mood. In this 12-week class-randomized trial, daily provision of an F&V-enriched breakfast did not produce significant between-group differences in global cognitive performance, parent-rated behavior, or mood compared with a nutritionally matched control breakfast. Notably, the intervention was successfully implemented and resulted in higher dietary fiber and carotene intake, indicating good feasibility and adherence in a school-based setting. Modest changes over time were observed in selected behavioral and mood domains, including exploratory age-specific patterns, although these changes were comparable between groups. Overall, the findings suggest that short-term enrichment of breakfast with fruits and vegetables alone may be insufficient to produce detectable between-group neuropsychological benefits within 12 weeks in school-aged children with relatively adequate baseline nutritional and cognitive status.

Observational studies have consistently linked higher F&V intake and overall diet quality with improved academic achievement, attention, and emotional regulation in children and adolescents [4,5,6]. However, randomized evidence remains limited and mixed, with short-term interventions often showing null or domain-specific effects rather than broad cognitive gains [16,17]. A recent synthesis likewise noted that benefits, when present, tend to be modest and confined to specific domains such as working memory [18]. In line with previous intervention studies, the present trial did not detect statistically significant between-group differences in global cognitive performance over the 12-week intervention period. Several cognitive indices nevertheless increased from baseline to week 12 in both the intervention and control groups. These parallel improvements may partly reflect practice effects associated with repeated administration of the WISC-IV, which are well documented in pediatric populations. In addition, participation in a structured breakfast program itself, irrespective of dietary composition, may have contributed to improved routine, engagement, or test familiarity [19]. It is also notable that children enrolled in this trial demonstrated relatively adequate baseline cognitive performance and dietary intake, which may have limited the potential for detecting additional cognitive benefits. At the same time, because FSIQ is a relatively stable construct, and because the dietary modification was limited to one breakfast component, a 12-week intervention may not be sufficiently intensive or long enough to produce detectable between-group differences in this endpoint. Overall, these design and measurement considerations provide plausible explanations for why F&V enrichment did not yield additional cognitive gains beyond the control breakfast in this trial.

Beyond cognition, diet quality has been associated with behavioral and emotional outcomes in children. Diets rich in refined carbohydrates and processed foods are associated with greater hyperactivity and emotional difficulties, whereas those emphasizing F&V and whole grains are associated with fewer behavioral problems [7,20,21]. In the present study, no significant between-group differences were detected in parent-rated behavioral outcomes. Nevertheless, modest changes over time were observed in the somatic symptoms, which might reflect improved satiety, more stable morning glycemia, or higher fiber intake, factors previously implicated in emotional and behavioral regulation [22]. Although these observations did not translate into significant between-group differences, they highlight behavioral domains that may be sensitive to dietary context and warrant further investigation in studies with longer duration or more intensive dietary contrasts.

Mood outcomes were largely unchanged, aligning with prior studies showing that increases in F&V consumption tend to enhance perceived energy rather than reduce negative mood states [5,23,24]. Meta-analyses further suggest that each 100 g increase in F&V intake is associated with a small but measurable reduction in depressive symptoms [25], possibly through improved nutrient availability for neurotransmitter synthesis and attenuation of low-grade inflammation [26]. Given that participants in our study were healthy children with low baseline symptom levels, substantial mood changes were unlikely to emerge over a 12-week period. Nevertheless, exploratory age-stratified analyses indicated a modest reduction in fatigue among children aged ≤8 years, suggesting that age-related differences in responsiveness may exist. This age-specific pattern may plausibly relate to greater sensitivity of younger children to short-term changes in breakfast quality, including dietary fiber-related improvements in morning energy regulation, which have been linked to glycemic stability in prior studies [27]. Supporting this notion of age-dependent sensitivity, previous research has reported that children aged 6–8 years may exhibit more pronounced neurobehavioral responses to higher-fiber breakfast interventions compared with older children, although these effects were observed in cognitive rather than fatigue-related outcomes [28]. In addition, early school-age years represent a period of rapid neurodevelopment, during which dietary inputs may exert more immediate effects on subjective energy levels and fatigue before manifesting as changes in more stable cognitive constructs [29,30]. Although exploratory in nature, this finding highlights fatigue as a potentially sensitive mood-related outcome and may inform the design of future hypothesis-driven trials with longer follow-up or age-targeted interventions.

An important contextual factor is the habitual breakfast pattern among Chinese school-aged children. Breakfast consumption is often irregular, and many students skip breakfast altogether. When breakfast is eaten, it typically consists of refined carbohydrate-based foods such as steamed buns, noodles, or rice, with minimal or no vegetables [31]. In addition, children commonly dislike foods rich in dietary fiber, including F&V, for reasons related to taste preference and food culture [32,33,34]. This traditional breakfast pattern contributes to low fiber and micronutrient intake in the first meal of the day in China. By integrating F&V into breakfast rather than snacks or lunch, our intervention directly addressed this nutritional gap, offering a culturally relevant and feasible strategy to improve morning dietary quality and potentially support better daily functioning. From an implementation perspective, acceptability, particularly for vegetables at breakfast, represents an important practical consideration. Although overall adherence was high, field feedback suggested heterogeneity in children’s willingness to consume vegetables, consistent with picky eating and taste preference. To support successful scale-up, future interventions may benefit from child-friendly preparation, gradual exposure, and family engagement to facilitate repeated tasting and routine formation.

Several strengths enhance the validity of this study. The class-randomized design reduced the risk of contamination between intervention and control groups within the school context. All breakfasts were centrally prepared according to standardized specifications with comparable energy provision, and were individually packaged and delivered directly to participants’ homes. Because breakfasts were typically consumed at home, the likelihood of food sharing between participants from different study arms was minimized. Nonetheless, several limitations should be acknowledged. The 12-week duration and the modest intervention intensity, limited to substituting the side component of one daily meal, may be insufficient to detect changes in stable neurocognitive outcomes such as FSIQ. Parent-reported questionnaires may also be less sensitive to subtle changes in attention and mood in generally healthy children with low baseline symptom levels. Although the delivery model reduced the risk of contamination, occasional sharing cannot be completely excluded. In addition, while breakfast was tightly standardized, the remainder of the daily diet was not controlled, and compensatory intake later in the day could have diluted effects despite dietary assessment efforts. The study’s single-site urban setting may limit generalizability, and some degree of cross-class contamination cannot be excluded despite class-level randomization. Moreover, objective academic data were not collected because individual student performance is treated as confidential under education privacy regulations. Future research should include longer follow-up periods, multiple schools with diverse dietary backgrounds, and more sensitive or objective measures such as teacher ratings, standardized cognitive assessments, or neuroimaging outcomes.

## 5. Conclusions

In conclusion, this 12-week F&V-enriched breakfast intervention was feasible within a school-linked research framework and successfully increased dietary fiber and carotene intake among children. No statistically significant between-group differences were observed in cognitive performance, attention-related behavioral symptoms, or mood outcomes. Exploratory analyses further suggested a modest reduction in fatigue among younger children in the intervention group. Overall, these findings support the feasibility of improving breakfast composition through a substitution approach, while suggesting that longer or more intensive interventions and more sensitive outcomes may be required to detect broader neurobehavioral effects.

## Figures and Tables

**Table 1:** Baseline characteristics of participants by intervention group (*n* = 251).

Characteristics	Intervention Group (*n* = 129)	Control Group (*n* = 122)
Age, (years)	8.5 (1.0)	8.7 (1.0)
Sex, *n* (%)		
Male	79 (61.2)	68 (55.7)
Female	50 (38.8)	54 (44.3)
BMI (kg/m^2^)	17.0 (3.6)	17.0 (3.0)
Waist (cm)	59.6 (10.9)	62.1 (8.8)
Hipline (cm)	71.8 (7.8)	72.8 (8.0)
Skinfold (cm)	19.5 (5.7)	19.2 (6.3)
Feeding Pattern (Infancy), *n* (%)		
Exclusive breastfeeding until 6 months	32 (24.8)	38 (31.1)
Exclusive breastfeeding until 12 months	47 (36.4)	31 (25.4)
Exclusive breastfeeding until 36 months	5 (3.9)	2 (1.6)
Mixed feeding (formula + breast milk)	37 (28.7)	37 (30.3)
Exclusive formula feeding	7 (5.4)	7 (5.7)
Other	1 (0.8)	7 (5.7)
Father’s Education Level, *n* (%)		
Junior high school or below	5 (3.9)	3 (2.5)
High school	18 (14.0)	11 (9.0)
Associate degree	26 (20.2)	31 (25.4)
Bachelor’s degree	74 (57.4)	68 (55.7)
Master’s degree or above	6 (4.7)	9 (7.4)
Mother’s Education Level, *n* (%)		
Junior high school or below	6 (4.7)	4 (3.3)
High school	14 (10.9)	14 (11.5)
Associate degree	31 (24.0)	36 (29.5)
Bachelor’s degree	71 (55.0)	62 (50.8)
Master’s degree or above	7 (5.4)	6 (4.9)
Income per Capital, *n* (%)		
<100 K	24 (18.6)	18 (14.8)
100–200 K	51 (39.5)	41 (33.6)
200–300 K	16 (12.4)	22 (18.0)
300–400 K	8 (6.2)	9 (7.4)
400–500 K	3 (2.3)	7 (5.7)
≥500 K	9 (7.0)	6 (4.9)
Not sure	18 (14.0)	19 (15.6)
Baseline Dietary intake		
Energy (kcal/day)	1429.54 (413.47)	1452.40 (368.24)
Protein (g/day)	72.03 (25.05)	71.91 (22.61)
Fat (g/day)	47.65 (24.63)	44.40 (15.47)
Carbohydrates (g/day)	191.79 (60.76)	201.03 (57.50)
Dietary Fiber (g/day)	15.81 (7.88)	15.96 (7.81)
Carotene (mg/day)	1.91 (1.96)	1.71 (1.39)
Baseline primary and secondary outcomes		
WISC-IV Full-Scale IQ	106.73 (15.34)	108.46 (15.31)
General Ability Index	109.11 (16.77)	110.27 (16.24)
Cognitive Proficiency Index	101.93 (15.67)	104.08 (16.02)
PSQ Total Score	22.69 (16.69)	22.19 (15.53)
POMS Total Mood Disturbance	95.67 (20.65)	96.62 (19.26)

Note: Data are presented as mean (SD) for continuous variables and *n* (%) for categorical variables and no formal statistical testing was performed at baseline. BMI, body mass index; WISC-IV, Chinese Wechsler Intelligence Scale for Children—Fourth Edition; PSQ, Parent Conners Symptom Questionnaire; POMS, Profile of Mood States—Brief; SD, standard deviation.

**Table 2:** Changes in primary and secondary outcomes from baseline to post-intervention using ITT analysis.

Outcome	Δ Intervention Group (Mean [SD])	Δ Control Group (Mean [SD])	Δ Mean Difference (95% CI)	*p* Value
No.	251	251		
Primary outcome				
WISC-IV Full-Scale IQ	2.33 (4.84)	2.96 (4.20)	−0.63 (−5.15~3.89)	0.807
Secondary outcomes (Cognitive)				
General Ability Index	2.82 (5.83)	2.43 (4.98)	0.38 (−4.84~5.60)	0.735
Cognitive Proficiency Index	7.96 (4.30)	7.83 (3.00)	0.13 (−5.46~5.72)	0.897
Secondary outcomes (Behavior & Mood)				
PSQ Total Score	−2.56 (1.64)	−2.38 (2.59)	−0.18 (−4.99~4.63)	0.968
POMS Total Mood Disturbance	−0.51 (2.20)	−1.11 (1.95)	0.60 (−4.86~6.06)	0.927

Note: Analyses were performed under the ITT framework using multiple imputation to handle missing data. Values are presented as mean change (SD) from baseline to post-intervention for each group. The Δ Mean difference column denotes the between-group difference in mean change, calculated as intervention minus control. All *p* values are derived from adjusted models controlling for baseline age, sex, BMI, and household income. SD, standard deviation; CI, confidence intervals; WISC-IV, Chinese Wechsler Intelligence Scale for Children—Fourth Edition; PSQ, Parent Conners Symptom Questionnaire; POMS, Profile of Mood States—Brief; ITT, intention-to-treat; BMI, body mass index.

**Table 3:** Pre- and post-intervention PSQ domains domain scores using ITT analysis.

Indicator	Intervention Group		Control Group		*p* Value
	Pre-Intervention	Post-Intervention	Pre-Intervention	Post-Intervention	
PSQ total score	22.69 (16.69)	19.58 (17.21)	22.19 (15.53)	18.96 (14.42)	0.583
Inattention	1.98 (1.54)	1.67 (1.55)	1.98 (1.43)	1.60 (1.38)	0.475
Hyperactivity/Impulsivity	2.38 (2.04)	1.88 (1.94)	2.05 (1.90)	1.92 (1.92)	0.266
Behavioral and Social Problems	4.79 (4.80)	4.18 (4.71)	4.43 (4.20)	3.98 (3.99)	0.621
Emotional Problems	4.13 (2.90)	3.54 (2.88)	4.31 (2.77)	3.50 (2.45)	0.519
Somatic Symptoms	1.53 (1.89)	1.10 (1.58)	1.68 (1.92)	1.22 (1.74)	0.626

Note: All statistical tests were performed under the ITT framework using multiple imputation to address missing data. Data are presented as mean (SD). *p* values correspond to between-group differences in change from baseline to post-intervention estimated under the ITT framework with multiple imputation. ITT, intention-to-treat; PSQ, Parent Conners Symptom Questionnaire; SD, standard deviation.

**Table 4:** Comparison of daily nutrient intakes between the intervention and control groups after the intervention.

Nutrient	Intervention Group	Control Group	*p* Value
Energy (kcal)	1166.01 (343.37)	1208.87 (308.99)	0.395
Protein (g)	67.85 (23.97)	67.45 (17.96)	0.901
Fat (g)	40.55 (16.27)	40.26 (12.80)	0.900
Carbohydrates (g)	143.56 (45.28)	152.00 (47.14)	0.235
Dietary Fiber (g)	15.80 (6.53)	13.68 (5.82)	0.027 *
Cholesterol (mg)	452.65 (220.67)	408.51 (185.37)	0.162
Ash (g)	11.06 (4.19)	10.85 (3.59)	0.733
Vitamin A (μg)	451.23 (241.90)	394.98 (259.75)	0.146
Carotene (mg)	2.04 (1.48)	1.58 (1.06)	0.023 *
Retinol (μg)	262.20 (175.46)	242.19 (205.67)	0.495
Thiamin (mg)	0.70 (0.26)	0.72 (0.22)	0.713
Riboflavin (mg)	0.91 (0.35)	0.84 (0.29)	0.197
Niacin (mg)	14.11 (4.82)	14.79 (4.60)	0.353
Vitamin C (mg)	81.15 (60.59)	67.85 (55.62)	0.139
Total Vitamin E (mg)	8.52 (5.93)	8.06 (5.15)	0.588
α-Vitamin E (mg)	3.31 (1.53)	3.27 (2.39)	0.895
(β + γ)-Vitamin E (mg)	2.86 (3.04)	2.48 (2.47)	0.384
δ-Vitamin E (mg)	1.06 (1.07)	1.13 (1.44)	0.723
Calcium (mg)	475.57 (212.83)	455.97 (166.61)	0.508
Phosphorus (mg)	871.23 (257.99)	871.56 (239.27)	0.993
Potassium (mg)	1806.14 (647.01)	1683.95 (549.70)	0.189
Sodium (mg)	898.91 (470.34)	893.75 (367.65)	0.937
Magnesium (mg)	194.91 (65.67)	191.59 (61.98)	0.736
Iron (mg)	13.98 (8.09)	12.64 (5.58)	0.215
Zinc (mg)	8.39 (2.75)	8.86 (3.59)	0.332
Selenium (μg)	33.89 (14.96)	33.09 (10.96)	0.693
Copper (mg)	1.19 (1.69)	1.06 (1.33)	0.565
Manganese (mg)	2.12 (1.12)	2.05 (1.23)	0.713

Note: Values are presented as mean (SD). *p* values were calculated using independent samples *t*-tests for between-group comparisons after the intervention. Symbols indicate statistical significance: * *p* < 0.05. SD, standard deviation.

## Data Availability

Data described in the manuscript and analytic code are available from the corresponding author on reasonable request. All requests will be reviewed to ensure appropriate use and compliance with ethical guidelines.

## Supplementary Materials

The following supporting information can be downloaded at: https://www.mdpi.com/article/10.3390/nu18040581/s1, Supplementary Methods; Table S1: Shared components provided to both arms (main dish and side dish 1 only); Table S2. Intervention-only components by day (vegetable side dish 2 and fruit); Table S3. Control-only component by day (alternative side dish 3); Table S4. Parental satisfaction survey conducted after the intervention; Table S5. Weekly breakfast frequency among participants at baseline/Frequency of consuming breakfast prepared outside the home at baseline; Table S6. Pre- and post-intervention comparison of cognitive, behavioral, and mood indicators between the intervention and control groups under the ITT framework; Table S7. ITT analysis of changes from baseline to post-intervention across cognitive, behavioral, and mood subscales; Table S8. Interaction effects of time and group on cognitive, behavioral, and mood indicators under the ITT framework; Table S9. Pre-intervention and follow-up comparison of cognitive, behavioral, and mood indicators between the intervention and control groups under the ITT framework; Table S10. Sensitivity analysis using inverse probability weighting for post-intervention outcomes; Table S11. PP analysis of changes in cognitive, behavioral, and mood indicators from baseline to post-intervention; Table S12. Comparison of daily nutrient intakes between the intervention and control groups before the intervention.

## Abbreviations

List of Abbreviations in alphabetical order.

ADHD	Attention-deficit/hyperactivity disorder
BMI	Body mass index
CI	Confidence intervals
F&V	Fruits and vegetables
FSIQ	Full-Scale IQ
IPW	Inverse-probability weighting
ITT	Intention-to-treat
POMS	Profile of Mood States—Brief
PP	Per-protocol
PSQ	Parent Conners Symptom Questionnaire
SD	Standard deviation
WHO	World Health Organization
WISC-IV	Wechsler Intelligence Scale for Children—Fourth Edition
