# Supplementary material for: Effects of a Fruit- and Vegetable-Enriched Breakfast on Cognition, Attention, and Mood in Primary School Children: A Randomized Controlled Trial
Source: Nutrients. 2026 Feb 10;18(4):581. doi: 10.3390/nu18040581 (PMC12943250; doi:10.3390/nu18040581)
Supplement: Supplementary file 1 [file nutrients-18-00581-s001.zip › nutrients-4136669-supplementary.pdf]

Supplementary Materials

Effects of a Fruit- and Vegetable-Enriched Breakfast on Cognition, Attention, and Mood in Primary School Children: A Randomized Controlled Trial

Authors: Wenyun Li [1,†], Xiaotian Du [2,†], Yuanwei Ma [3], Huliang Cao [4], Shan Jin [2], Jing Fan [2], Jian Gao [1], Min Hou [3,*] and Bo Chen [2,*]

1 Department of Clinical Nutrition, Zhongshan Hospital, Fudan University, Shanghai 200032, China;

2 Department of Nutrition & Food Hygiene, School of Public Health, Key Laboratory of Public Health Safety of the Ministry of Education, Fudan University, Shanghai 200032, China;

3 School of Public Health, College of Medicine, Shanghai Jiao Tong University, Shanghai 200025, China;

4 Shanghai Food Industry Institute, Shanghai 200090, China; caohuliang@126.com

* Correspondence: minhou@sjtu.edu.cn (M.H.); chenb@fudan.edu.cn (B.C.)

† These authors contributed equally to this work.

Table of Contents

Supplementary Methods. 3

Supplementary Table S1. Shared components provided to both arms (main dish and side dish 1 only) 4

Supplementary Table S1A. Standard portion specifications of shared components 5

Supplementary Table S1B. Item-to-portion mapping for side dish 1 (30~35 g exceptions) 5

Supplementary Table S2. Intervention-only components by day (vegetable side dish 2 and fruit) 6

Supplementary Table S3. Control-only component by day (alternative side dish 3) 7

Supplementary Table S4. Parental satisfaction survey conducted after the intervention 8

Supplementary Table S5A. Weekly breakfast frequency among participants at baseline 8

Supplementary Table S5B. Frequency of consuming breakfast prepared outside the home at baseline 8

Supplementary Table S6. Pre- and post-intervention comparison of cognitive, behavioral, and mood indicators between the intervention and control groups under the ITT framework 9

Supplementary Table S7. ITT analysis of changes from baseline to post-intervention across cognitive, behavioral, and mood subscales 10

Supplementary Table S8. Interaction effects of time and group on cognitive, behavioral, and mood indicators under the ITT framework 11

Supplementary Table S9. Pre-intervention and follow-up comparison of cognitive, behavioral, and mood indicators between the intervention and control groups under the ITT framework 12

Supplementary Table S10. Sensitivity analysis using inverse probability weighting for post-intervention outcomes 13

Supplementary Table S11. PP analysis of changes in cognitive, behavioral, and mood indicators from baseline to post-intervention 14

Supplementary Table S12. Comparison of daily nutrient intakes between the intervention and control groups before the intervention 15

Supplementary Methods.

Assessor training

All cognitive, attention, and mood assessments were conducted by graduate-level staff with backgrounds in psychology, education, or nutrition. Before fieldwork, assessors completed centralized training that included didactic instruction on each instrument, mock assessments, and supervised pilot testing. Inter-rater reliability was evaluated against gold-standard scoring on 20 trial assessments, with ≥0.90 agreement required for certification. Ongoing calibration meetings were held biweekly to resolve ambiguities and prevent drift.

Assessment environment

To minimize distraction, cognitive and questionnaire-based assessments were conducted in quiet classrooms at school or in designated household settings during home visits. Rooms were arranged to reduce visual and auditory disturbance, with only the assessor and child present (a parent remained nearby if needed for assent or questionnaires). Background noise, mobile phones, and interruptions were restricted.

Standardization of procedures

Assessments followed a fixed order: the Chinese Wechsler Intelligence Scale for Children- Fourth Edition (WISC-IV) cognitive testing, followed by the Chinese Parent Conners Symptom Questionnaire (PSQ) and Profile of Mood States- Brie (POMS) questionnaires, then anthropometry and dietary recalls. Sessions lasted ~90-120 minutes, with breaks scheduled to reduce fatigue. Assessors used scripted introductions and standardized instructions to ensure uniform delivery. For home visits, portable equipment and standardized materials were used to replicate classroom conditions.

Field organization and quality control

The study team coordinated with teachers to schedule data collection without disrupting academic activities. Field supervisors observed ~10% of sessions unannounced to monitor fidelity. Completed forms were checked the same day and double-entered into a secure database with automated range and logic checks. Outliers (>3 SD) were verified against source records before database lock.

Menu rotation and portion standards

Breakfast menus were developed by nutritionists in consultation with parents and adjusted seasonally to ensure acceptability, freshness, and cultural appropriateness. Parental input was gathered in two pre-trial meetings and via weekly feedback during implementation; item-level changes were made when acceptability thresholds were not met. While the overall structure of meals was standardized (staple, egg item, milk, and arm-specific side dishes), the specific fruit, vegetable, and snack items varied across the intervention period in response to seasonal availability and parental feedback.

To illustrate the rotation, Supplementary Tables S1-S3 present menus from November 2024 as a representative example. Supplementary Table S1 details portion standards for shared components and small-item exceptions; Supplementary Table S2 lists intervention-only items (fruits and vegetables), and Supplementary Table S3 lists control-only items (snacks). Any deviations from standard portions, as documented during procurement or delivery, were incorporated into nutrient estimates following study SOPs.

Sample size calculation

The primary outcome was change in the Full Scale IQ (FSIQ) from the WISC-IV. The Chinese version of the WISC-IV has been widely validated in local populations and applied in intervention studies.

For sample size estimation, we referred to published pediatric cognitive intervention trials reporting small-to-moderate improvements in standardized IQ scores (Wu et al., 2023). In that study, the mean FSIQ change was 3.28 points in the intervention group and 1.11 points in the control group, yielding a between-group difference of 2.17 points. Using the maximum reported standard deviation ($\sigma = 4.11$) as a conservative variance estimate, we applied the two-sample mean comparison formula:

$$n = [(Z\alpha/2 + Z\beta)^2 \times 2\sigma^2] / (\mu_1 - \mu_2)^2 = 75$$

Substituting the values ($\alpha = 0.05$, power = 0.80), the required sample size was 75 per arm (150 total). Allowing for 20% attrition across repeated assessments, the minimum required sample size was 188.

In practice, recruitment was organized at the grade level to ensure fairness and acceptability: restricting enrollment to selected classes would have raised concerns among families and complicated logistics. Therefore, 251 children were enrolled, which exceeded the minimum requirement and provided additional precision for prespecified secondary and exploratory analyses.

Supplementary Table S1. Shared components provided to both arms (main dish and side dish 1 only)

Date	Main dish	Ingredients	Side dish 1
Day1	Braised Beef	Beef Shank	Mushroom & Veggie Xiaolongbao
Day2	Braised Pork with Mushroom	Pork, Seafood Mushroom	Paper-Wrapped Shrimp Dumpling
Day3	Fried Shrimp with Garlic	Shrimp	Beef Dumpling
Day4	Sauced Duck Breast	Duck Breast	Mini Pork Bun
Day5	Stir-Fried Beef with Scallion	Beef	Three Delicacies Shumai
Day6	Pumpkin Millet Red Date Porridge	Millet, Pumpkin, Red Dates	Shrimp Dumpling + Purple Yam Roll
Day7	Chicken & Mushroom Porridge	Chicken, Mushroom, Carrot, Greens	Paper-Wrapped Shumai
Day8	Purple Sweet Potato & Corn Porridge	Purple Sweet Potato, Corn	Pork Xiaolongbao
Day9	Radish & Pork Porridge	Radish, Carrot, Pork	Three Delicacies Shumai
Day10	Red Bean & Yam Porridge	Red Bean, Yam	Corn & Pork Dumpling
Day11	Century Egg & Pork Porridge	Century Egg, Pork, Greens	Red Bean Roll
Day12	Eight Treasure Porridge	Century Egg, Pork, Greens	Mushroom & Veggie Xiaolongbao
Day13	Shrimp & Veggie Porridge	Shrimp, Lettuce	Paper-Wrapped Shrimp Dumpling
Day14	Yam & Red Date Millet Porridge	Millet, Yam, Red Dates, Goji	Mini Pork Bun
Day15	Salted Egg & Pork Porridge	Salted Egg, Pork, Peas, Scallion	Beef Dumpling
Day16	Sweet & Sour Pork with Pineapple	Pork, Pineapple	Scallion Flower Roll
Day17	Pumpkin Millet Red Date Porridge	Millet, Pumpkin, Red Dates	Beef Dumpling
Day18	Chicken & Mushroom Porridge	Chicken, Mushroom	Custard Bun
Day19	Steamed Chicken	Chicken	Three Delicacies Shumai
Day20	Century Egg & Pork Porridge	Century Egg, Pork, Greens	Purple Yam Roll
Day21	Red Bean & Oat Porridge	Red Rice, Oats, Red Bean	Pork Bun
Day22	Tomato Fish Fillet	Fish Fillet, Tomato	Paper-Wrapped Shumai
Day23	Greens & Pork Porridge	Greens, Pork	Red Bean Roll
Day24	Purple Sweet Potato & Corn Porridge	Purple Sweet Potato, Corn Kernels	Three Delicacies Steamed Dumpling
Day25	Steamed Pork with Taro	Pork Ribs, Taro	Mushroom & Veggie Xiaolongbao
Day26	Pumpkin Millet Red Date Porridge	Millet, Pumpkin, Red Dates	Beef Dumpling
Day27	Chicken & Mushroom Porridge	Chicken, Mushroom	Scallion Flower Roll
Day28	Salted Beef	Beef	Pumpkin Pancake
Day29	Century Egg & Pork Porridge	Century Egg, Pork, Greens	Mushroom & Veggie Xiaolongbao
Day30	Red Bean & Oat Porridge	Red Rice, Oats, Red Bean	Paper-Wrapped Shumai

Note: This table lists only the day-by-day main dish and side dish 1 shared by both arms; weights are intentionally omitted for readability. Standard portions for shared components are defined in Supplementary Table S1a. Item-level exceptions for side dish 1 (< 50 g items) are enumerated in Supplementary Table S1b. The egg item and milk were fixed daily in both arms and are omitted here for brevity; see S1a for their standard portions. Menus from November 2024 are presented as a representative example; while the overall structure was consistent throughout the trial, specific items were adjusted seasonally and according to

parental feedback.

Supplementary Table S1A. Standard portion specifications of shared components

Component (shared)	Portion rule	Edible-portion	Prep/notes
Main dish: porridge	1 bowl ≈ 300 g	After cooking, edible portion	Fixed recipe per kitchen SOP
Main dish: protein/vegetable dish	50 g	After cooking, edible portion	Fixed recipe per kitchen SOP
Side dish 1: items listed in S1b	30~35 g	After cooking, edible portion	Small-size dim sum; see S1b list
Side dish 1: all other items	50 g	After cooking, edible portion	Default standard for side dish 1
Egg item	50 g	Whole, peeled	Rotating types; constant portion
Milk	200 mL	As served	Plain milk

Note: Masses in g; volumes in mL. Portions refer to edible portion as served; recipes and yields followed standardized kitchen SOPs.

Supplementary Table S1B. Item-to-portion mapping for side dish 1 (30~35 g exceptions)

Side dish 1 item name	Standard portion (g)	Notes
Mushroom & Veggie Xiaolongbao	35	
Paper-Wrapped Shrimp Dumpling	35	
Mini Pork Bun	35	Mini-size item
Paper-Wrapped Shumai	30	
Pork Xiaolongbao	35	
Corn & Pork Dumpling	30	
Red Bean Roll	35	
All other side dish 1 items	50	Default standard

Note: This table lists the only side dish 1 items assigned to 30 g or 35 g; all other side dish 1 items are 50 g. Edible portion after cooking; preparation per SOP.

Supplementary Table S2. Intervention-only components by day (vegetable side dish 2 and fruit)

Date	Vegetable side (side dish 2)	Fruit
Day1	Stir-Fried Cabbage with Fungus	Kiwi
Day2	Stir-Fried Potato & Onion	Red Dragon Fruit
Day3	Stir-Fried Mushroom & Greens	Kiwi
Day4	Lotus Root Patty	Cantaloupe
Day5	Stir-Fried Cabbage & Tofu Puff	Kiwi
Day6	Stir-Fried Lettuce	Cantaloupe
Day7	Tomato & Winter Melon Cubes	Kiwi
Day8	Stir-Fried Cabbage with Fungus	Cherry Tomato
Day9	Stir-Fried Potato & Onion	Kiwi
Day10	Stir-Fried Spinach	Blueberry
Day11	Stir-Fried Broccoli	Kiwi
Day12	Stir-Fried Cucumber & Yam	Red Dragon Fruit
Day13	Three-Color Veggie Stir-Fry	Kiwi
Day14	Baby Cabbage & Tofu Puff Stir-Fry	Orange
Day15	Lotus Root & Veggie Stir-Fry	Kiwi
Day16	Stir-Fried Mushroom & Greens	Cantaloupe
Day17	Mixed Veggie & Winter Melon	Kiwi
Day18	Baby Cabbage	Cherry Tomato
Day19	Tomato & Cucumber Stir-Fry	Kiwi
Day20	Stir-Fried Spinach	Blueberry
Day21	Stir-Fried Asparagus & Fungus	Kiwi
Day22	Stir-Fried Cabbage & Vermicelli	Red Dragon Fruit
Day23	Lotus Root & Veggie Stir-Fry	Kiwi
Day24	Oyster Sauce Broccoli	Orange
Day25	Two-Color Potato Shreds	Kiwi
Day26	Stir-Fried Mushroom & Greens	Cantaloupe
Day27	Mixed Veggie & Winter Melon	Kiwi
Day28	Baby Cabbage	Cherry Tomato
Day29	Tomato & Cucumber Stir-Fry	Kiwi
Day30	Stir-Fried Bok Choy	Hainan Melon

Note: Nutrient calculations used fixed standard portions: vegetable side dish 2 ≈ 60 g per serving; fruit ≈ 80 g per serving. Any deviations, if present, were handled per SOP and reflected in nutrient calculations. Menus from November 2024 are presented as a representative example; while the overall structure was consistent throughout the trial, specific items were adjusted seasonally and according to parental feedback.

Supplementary Table S3. Control-only component by day (alternative side dish 3)

Date	Side dish 3	Date	Side dish 3
Day1	Popcorn	Day16	Purple Sweet Potato
Day2	Almond	Day17	Popcorn
Day3	Sweet Potato	Day18	Taro
Day4	Mashed Potato	Day19	Salt & Pepper Fries
Day5	Walnut	Day20	Yam
Day6	Cashew	Day21	Pumpkin
Day7	Corn Cob	Day22	Mashed Potato
Day8	Pumpkin	Day23	Sweet Potato
Day9	Macadamia Nut	Day24	Crispy Rice Cake
Day10	Sweet Potato	Day25	Corn Cob
Day11	Salt & Pepper Potato	Day26	Popcorn
Day12	Almond	Day27	Purple Sweet Potato
Day13	Yam	Day28	Seaweed Stem
Day14	Purple Sweet Potato	Day29	Salt & Pepper Fries
Day15	Walnut	Day30	Yam

Note: Nutrient calculations used a fixed standard portion: side dish 3 ≈ 40 g per serving. Any deviations, if present, were handled per SOP and reflected in nutrient calculations. Menus from November 2024 are presented as a representative example; while the overall structure was consistent throughout the trial, specific items were adjusted seasonally and according to parental feedback.

Supplementary Table S4. Parental satisfaction survey conducted after the intervention

Variables	Intervention group	Control group	Total	*P* value
Physical Discomfort During Program				0.638
NO	116 (89.9)	102 (83.6)	218 (86.9)	
YES	9 (7.0)	11 (9.0)	20 (8.0)	
Satisfaction Level				0.663
Very satisfied	57 (44.2)	54 (44.3)	111 (44.2)	
Mostly satisfied	49 (38.0)	38 (31.1)	87 (34.7)	
Neutral	17 (13.2)	20 (16.4)	37 (14.7)	
Mostly dissatisfied	2 (1.6)	1 (0.8)	3 (1.2)	
Very dissatisfied	0 (0)	0 (0)	0 (0)	

Note: Values are n (%).

Supplementary Table S5A. Weekly breakfast frequency among participants at baseline

Variables	Intervention group	Control group	Total	*P* value 1	*P* value 2
Every day	112 (86.8)	100 (82.0)	212 (84.5)	0.159	0.302
5-6 times	9 (7.0)	18 (14.8)	27 (10.8)		
3-4 times	5 (3.9)	4 (3.3)	9 (3.6)		
1-2 times	2 (1.6)	0 (0)	2 (0.8)		
Rarely / Almost never	1 (0.8)	0 (0)	1 (0.4)		

Note: Values are n (%). *P* value 1 was derived from a Pearson chi-square test comparing the distribution of weekly breakfast frequency between the intervention and control groups. *P* value 2 was derived from a Fisher's exact test performed on a dichotomized comparison (every day versus less than every day).

Supplementary Table S5B. Frequency of consuming breakfast prepared outside the home at baseline

Frequency category	Intervention group	Control group	Total	*P* value1	*P* value2
Never	61 (24.4)	64 (25.6)	125 (49.8)	0.478	0.448
1-2 times per week	52 (20.8)	42 (16.8)	94 (37.5)		
3-4 times per week	6 (2.4)	9 (3.6)	15 (6.0)		
5-7 times per week	10 (4.0)	6 (2.4)	16 (6.4)		

Note: Values are n (%). Prepared outside includes restaurant dine-in, takeout, or delivery, excluding school canteens. *P* value 1 was derived from a Pearson chi-square test comparing the distribution of outside-prepared breakfast frequency between the intervention and control groups. *P* value 2 was derived from a Fisher's exact test performed on a dichotomized comparison (never versus ever).

Supplementary Table S6. Pre- and post-intervention comparison of cognitive, behavioral, and mood indicators between the intervention and control groups under the ITT framework

Indicator	Intervention group			Control group			*P* value 2
	Pre-intervention	Post-intervention	*P* value 1	Pre-intervention	Post-intervention	*P* value 1	
Primary outcome							
WISC-IV (Cognitive performance)							
Full Scale IQ	106.73 (15.34)	115.98 (15.80)	0.194	108.46 (15.31)	117.90 (17.21)	0.220	0.470
Secondary outcomes							
WISC-IV subscales (Cognitive)							
Verbal Comprehension	114.58 (19.96)	118.13 (19.35)	0.119	115.15 (18.48)	120.15 (19.01)	0.285	0.649
Perceptual Reasoning	100.41 (15.19)	108.34 (15.57)	0.218	100.80 (14.45)	107.88 (14.77)	0.214	0.640
Working Memory	100.56 (16.45)	105.81 (13.89)	0.203	102.07 (17.50)	108.04 (15.82)	0.186	0.556
Processing Speed	102.21 (17.83)	114.70 (17.90)	0.112	103.25 (18.66)	116.89 (20.83)	0.030	0.465
General Ability Index	109.11 (16.77)	115.99 (17.45)	0.165	110.27 (16.24)	116.79 (17.81)	0.194	0.693
Cognitive Proficiency Index	101.93 (15.67)	112.32 (15.01)	0.049	104.08 (16.02)	114.95 (16.36)	0.003	0.515
PSQ subscales (Behavioral)							
PSQ total score	22.69 (16.69)	19.58 (17.21)	0.124	22.19 (15.53)	18.96 (14.42)	0.339	0.580
Inattention	1.98 (1.54)	1.67 (1.55)	0.064	1.98 (1.43)	1.60 (1.38)	0.217	0.513
Hyperactivity/Impulsivity	2.38 (2.04)	1.88 (1.94)	0.081	2.05 (1.90)	1.92 (1.92)	0.181	0.603
Behavioral and Social Problems	4.79 (4.80)	4.18 (4.71)	0.432	4.43 (4.20)	3.98 (3.99)	0.205	0.569
Emotional Problems	4.13 (2.90)	3.54 (2.88)	0.050	4.31 (2.77)	3.50 (2.45)	0.072	0.577
Somatic Symptoms	1.53 (1.89)	1.10 (1.58)	0.047	1.68 (1.92)	1.22 (1.74)	0.243	0.516
POMS subscales (Mood)							
Total Mood Disturbance	95.67 (20.65)	95.73 (21.34)	0.751	96.62 (19.26)	95.64 (20.49)	0.435	0.874
Tension	4.23 (3.35)	3.90 (4.03)	0.303	4.29 (3.74)	4.18 (3.65)	0.554	0.555
Anger	3.01 (4.85)	3.30 (5.91)	0.670	3.36 (5.47)	3.04 (5.05)	0.464	0.723
Fatigue	3.56 (4.53)	3.23 (4.28)	0.377	3.34 (3.85)	3.42 (4.08)	0.716	0.651
Depression	2.27 (3.66)	2.33 (3.92)	0.706	2.59 (3.63)	2.38 (3.48)	0.426	0.786
Vigor	12.72 (6.34)	12.90 (6.30)	0.691	12.70 (6.18)	12.66 (6.37)	0.769	0.810
Confusion	3.22 (2.78)	3.36 (3.34)	0.736	3.68 (2.81)	3.11 (3.36)	0.144	0.645
Esteem-Related Affect	7.90 (4.56)	7.49 (4.66)	0.442	7.96 (3.96)	7.84 (4.30)	0.696	0.403

Note: All statistical tests were performed under the ITT framework using multiple imputation to address missing data. Data are presented as mean (SD) based on observed values. *P* value 1 denotes within-group comparisons (paired t-tests between pre- and post-intervention). *P* value 2 denotes between-group comparisons after the intervention (independent t-tests). ITT, intention-to-treat; WISC-IV, Chinese Wechsler Intelligence Scale for Children- Fourth Edition; POMS, Profile of Mood States- Brief; PSQ, Parent Conners Symptom Questionnaire; SD, standard deviation.

Supplementary Table S7. ITT analysis of changes from baseline to post-intervention across cognitive, behavioral, and mood subscales

Indicator	Δ Intervention group (mean [SD])	Δ Control group (mean [SD])	Δ Mean difference (95% CI)	*P* value
Cognitive (WISC-IV)				
Verbal Comprehension Index	0.41 (5.20)	0.99 (3.98)	0.58 (-5.71~6.87)	0.910
Perceptual Reasoning Index	4.10 (4.88)	3.54 (3.97)	-0.56 (-6.24~5.11)	0.692
Working Memory Index	3.86 (5.48)	4.20 (4.16)	0.34 (-5.64~6.31)	0.815
Processing Speed Index	8.57 (6.64)	9.31 (5.16)	0.74 (-7.04~8.52)	0.768
Behavioral (PSQ)				
Inattention	-0.29 (0.16)	-0.34 (0.26)	-0.04 (-0.51~0.43)	0.899
Hyperactivity/Impulsivity	-0.39 (0.22)	-0.05 (0.34)	0.34 (-0.27~0.95)	0.292
Behavioral and Social Problems	-0.39 (0.50)	-0.15 (0.77)	0.24 (-1.13~1.60)	0.792
Emotional Problems	-0.60 (0.29)	-0.84 (0.45)	-0.24 (-1.07~0.58)	0.599
Somatic Symptoms	-0.38 (0.19)	-0.36 (0.31)	0.02 (-0.55~0.59)	0.871
Mood (POMS)				
Tension	-0.45 (0.44)	-0.17 (0.43)	0.28 (-0.86~1.41)	0.481
Anger	0.19 (0.63)	-0.19 (0.68)	-0.38 (-2.02~1.26)	0.716
Fatigue	-0.38 (0.44)	0.04 (0.45)	0.42 (-0.73~1.58)	0.384
Depression	-0.07 (0.48)	-0.15 (0.49)	-0.07 (-1.27~1.12)	0.999
Vigor	0.22 (0.58)	0.11 (0.64)	-0.11 (-1.77~1.56)	0.921
Confusion	0.07 (0.37)	-0.49 (0.37)	-0.56 (-1.52~0.39)	0.286
Esteem-Related Affect	-0.36 (0.47)	0.06 (0.48)	0.42 (-0.82~1.66)	0.475

Note: Analyses were performed under the ITT framework using multiple imputation to handle missing data. Values are presented as mean change (SD) from baseline to post-intervention for each group. The Δ Mean difference column represents the between-group difference in mean change (intervention minus control). All *p* values are derived from adjusted models controlling for baseline age, sex, BMI, and household income. ITT, intention-to-treat; SD, standard deviation; CI, confidence intervals; WISC-IV, Chinese Wechsler Intelligence Scale for Children- Fourth Edition; PSQ, Parent Conners Symptom Questionnaire; POMS, Profile of Mood States- Brief; BMI, body mass index.

Supplementary Table S8. Interaction effects of time and group on cognitive, behavioral, and mood indicators under the ITT framework

Indicator	Pre- vs post-intervention		Pre-intervention vs follow-up	
	β (95% CI)	*P* value	β (95% CI)	*P* value
Primary outcome				
WISC-IV (Cognitive performance)				
Full Scale IQ	0.63 (-3.88~5.14)	0.784	0.67 (-4.13~5.48)	0.783
Secondary outcomes				
WISC-IV subscales (Cognitive)				
Verbal Comprehension	0.58 (-5.70~6.86)	0.856	-4.97 (-49.29~39.35)	0.823
Perceptual Reasoning	-0.56 (-6.23~5.11)	0.845	0.59 (-4.63~5.81)	0.826
Working Memory	0.34 (-5.63~6.30)	0.912	0.72 (-5.18~6.62)	0.811
Processing Speed	0.74 (-7.02~8.50)	0.851	-1.07 (-8.34~6.20)	0.773
General Ability Index	-0.38 (-5.60~4.83)	0.885	1.39 (-3.97~6.74)	0.611
Cognitive Proficiency Index	-0.13 (-5.72~5.46)	0.963	-0.89 (-6.07~4.29)	0.737
PSQ subscales (Behavioral)				
PSQ total score	0.18 (-4.62~4.98)	0.942	0.40 (-3.44~4.24)	0.839
Inattention	-0.04 (-0.51~0.43)	0.863	-0.06 (-0.41~0.30)	0.752
Hyperactivity/Impulsivity	0.34 (-0.27~0.95)	0.275	0.19 (-0.34~0.71)	0.486
Behavioral and Social Problems	0.24 (-1.12~1.60)	0.733	0.40 (-0.78~1.59)	0.506
Emotional Problems	-0.24 (-1.06~0.58)	0.561	-0.14 (-0.93~0.64)	0.723
Somatic Symptoms	0.02 (-0.55~0.59)	0.951	-0.19 (-0.66~0.29)	0.444
POMS subscales (Mood)				
Total Mood Disturbance	-0.60 (-6.07~4.88)	0.830	-2.36 (-8.28~3.56)	0.435
Tension	0.28 (-0.86~1.42)	0.634	0.21 (-1.14~1.56)	0.761
Anger	-0.38 (-2.02~1.26)	0.652	-0.56 (-2.48~1.37)	0.570
Fatigue	0.42 (-0.73~1.58)	0.472	0.39 (-0.93~1.70)	0.565
Depression	-0.07 (-1.27~1.12)	0.905	-0.37 (-1.77~1.03)	0.604
Vigor	-0.11 (-1.77~1.56)	0.900	1.12 (-0.89~3.12)	0.275
Confusion	-0.56 (-1.52~0.39)	0.249	-0.21 (-1.44~1.01)	0.733
Esteem-Related Affect	0.42 (-0.82~1.66)	0.504	0.80 (-0.63~2.24)	0.270

Note: Estimates (β) and 95% CI were obtained from LMMs testing the interaction between time (pre- versus post-intervention) and group (intervention versus control). All models included a random intercept for participant ID to account for within-subject correlations and were adjusted for baseline age, sex, BMI, and household income under the ITT framework with multiple imputation for missing data. ITT, intention-to-treat; WISC-IV, Chinese Wechsler Intelligence Scale for Children- Fourth Edition; POMS, Profile of Mood States- Brief; PSQ, Parent Conners Symptom Questionnaire; CI, confidence intervals; LMMs, linear mixed-effects models; BMI, body mass index.

Supplementary Table S9. Pre-intervention and follow-up comparison of cognitive, behavioral, and mood indicators between the intervention and control groups under the ITT framework

Indicator	Intervention group			Control group			*P* value 2
	Pre-intervention	Follow-up	*P* value 1	Pre-intervention	Follow-up	*P* value 1	
Primary outcome							
WISC-IV (Cognitive performance)							
Full Scale IQ	106.73 (15.34)	110.10 (15.89)	0.071	108.46 (15.31)	112.29 (16.20)	0.109	0.486
Secondary outcomes							
WISC-IV subscales (Cognitive)							
Verbal Comprehension	114.58 (19.96)	119.53 (94.61)	0.547	115.15 (18.48)	114.38 (18.98)	0.194	0.597
Perceptual Reasoning	100.41 (15.19)	108.08 (15.99)	0.183	100.80 (14.45)	109.34 (14.80)	0.085	0.681
Working Memory	100.56 (16.45)	105.05 (14.84)	0.148	102.07 (17.50)	106.92 (14.77)	0.103	0.506
Processing Speed	102.21 (17.83)	111.48 (20.45)	0.252	103.25 (18.66)	111.38 (20.17)	0.292	0.699
General Ability Index	109.11 (16.77)	112.20 (17.94)	0.066	110.27 (16.24)	115.31 (17.58)	0.127	0.368
Cognitive Proficiency Index	101.93 (15.67)	110.33 (15.40)	0.051	104.08 (16.02)	111.25 (15.75)	0.053	0.607
PSQ subscales (Behavioral)							
PSQ total score	22.69 (16.69)	17.90 (15.10)	0.403	22.19 (15.53)	18.0 (14.2)	0.315	0.829
Inattention	1.98 (1.54)	1.56 (1.34)	0.176	1.98 (1.43)	1.51 (1.27)	0.122	0.808
Hyperactivity/Impulsivity	2.38 (2.04)	1.78 (1.76)	0.378	2.05 (1.90)	1.71 (1.65)	0.329	0.761
Behavioral and Social Problems	4.79 (4.80)	3.79 (4.18)	0.306	4.43 (4.20)	3.91 (3.71)	0.076	0.736
Emotional Problems	4.13 (2.90)	3.27 (2.68)	0.415	4.31 (2.77)	3.31 (2.56)	0.314	0.825
Somatic Symptoms	1.53 (1.89)	1.04 (1.34)	0.270	1.68 (1.92)	1.00 (1.30)	0.085	0.805
POMS subscales (Mood)							
Total Mood Disturbance	95.67 (20.65)	94.50 (18.80)	0.452	96.62 (19.26)	93.6 (22.6)	0.430	0.575
Tension	4.23 (3.35)	3.19 (3.57)	0.207	4.29 (3.74)	3.58 (3.76)	0.302	0.564
Anger	3.01 (4.85)	3.03 (4.87)	0.290	3.36 (5.47)	3.14 (5.84)	0.330	0.660
Fatigue	3.56 (4.53)	2.36 (3.58)	0.242	3.34 (3.85)	2.85 (4.06)	0.305	0.617
Depression	2.27 (3.66)	2.34 (3.81)	0.332	2.59 (3.63)	2.28 (4.13)	0.173	0.726
Vigor	12.72 (6.34)	12.2 (6.34)	0.189	12.70 (6.18)	13.4 (6.33)	0.293	0.253
Confusion	3.22 (2.78)	2.78 (3.32)	0.125	3.68 (2.81)	3.19 (3.67)	0.233	0.702
Esteem-Related Affect	7.90 (4.56)	6.92 (4.72)	0.454	7.96 (3.96)	8.05 (4.65)	0.210	0.196

Note: All statistical tests were performed under the ITT framework using multiple imputation to address missing data. Values are presented as mean (SD) based on observed values. *P* value 1 denotes within-group comparisons (paired t-tests between pre- and post-intervention). *P* value 2 denotes between-group comparisons after the follow-up period (independent t-tests). ITT, intention-to-treat; WISC-IV, Chinese Wechsler Intelligence Scale for Children- Fourth Edition; POMS, Profile of Mood States- Brief; PSQ, Parent Conners Symptom Questionnaire; SD, standard deviation.

Supplementary Table S10. Sensitivity analysis using inverse probability weighting for post-intervention outcomes

Indicator	n/N	β (95% CI)	*P* value
Primary outcome			
WISC-IV (Cognitive performance)			
Full Scale IQ	121/251	5.46 (-0.97,11.90)	0.096
Secondary outcomes			
WISC-IV subscales (Cognitive)			
Verbal Comprehension	121/251	9.73 (2.20.17.20)	0.011
Perceptual Reasoning	121/251	0.83 (-5.01,6.68)	0.780
Working Memory	121/251	3.58 (-1.92,9.08)	0.202
Processing Speed	121/251	-0.04 (-7.40,7.33)	0.993
General Ability Index	121/251	6.27 (-0.68,13.20)	0.077
Cognitive Proficiency Index	121/251	2.27 (-3.86,8.40)	0.468
PSQ subscales (Behavioral)			
PSQ total score	135/251	-0.93 (-6.89,5.03)	0.760
Inattention	135/251	0.03 (-0.49,0.55)	0.910
Hyperactivity/Impulsivity	135/251	-0.07 (-0.75,0.62)	0.853
Behavioral and Social Problems	135/251	-0.71 (-2.38,0.95)	0.401
Emotional Problems	135/251	0.19 (-0.85,1.23)	0.720
Somatic Symptoms	135/251	0.05 (-0.59,0.70)	0.868
POMS subscales (Mood)			
Total Mood Disturbance	135/251	4.44 (-1.95,10.80)	0.173
Tension	135/251	1.35 (0.25,2.44)	0.016
Anger	135/251	0.47 (-1,05,1.98)	0.546
Fatigue	135/251	0.81 (-0.46,2.09)	0.210
Depression	135/251	0.83 (-0.17,1.84)	0.105
Vigor	135/251	-0.80 (-3.16,1.55)	0.503
Confusion	135/251	0.46 (-0.50,1.43)	0.346
Esteem-Related Affect	135/251	0.29 (-1.09,1.67)	0.683

Note: Estimates (β) and 95% CI were obtained from IPW models among participants who adhered to the assigned intervention. IPW was applied to adjust for potential selection bias due to non-random attrition or differential adherence. Models were adjusted for baseline age, sex, BMI, and household income under the ITT framework with multiple imputation for missing data. Number of observations/total number of participants (excluding non-compliers). CI, confidence intervals; WISC-IV, Chinese Wechsler Intelligence Scale for Children- Fourth Edition; PSQ, Parent Conners Symptom Questionnaire; POMS, Profile of Mood States- Brief; IPW, inverse probability weighting; BMI, body mass index; ITT, intention-to-treat.

Supplementary Table S11. PP analysis of changes in cognitive, behavioral, and mood indicators from baseline to post-intervention

Outcome	n_I/n_c	Intervention group	Control group	Mean difference (95% CI)	*P* value
Primary outcome					
WISC-IV (Cognitive performance)					
Full Scale IQ	101/97	3.39 (1.11)	3.02 (1.18)	0.36 (-2.83, 3.56)	0.807
Secondary outcomes					
WISC-IV subscales (Cognitive)					
Verbal Comprehension	101/97	0.66 (1.47)	0.90 (1.52)	-0.24 (-4.4, 3.93)	0.461
Perceptual Reasoning	101/98	5.21 (1.44)	4.34 (1.32)	0.86 (-2.99, 4.71)	0.886
Working Memory	101/98	2.20 (1.39)	2.13 (1.37)	0.07 (-3.78, 3.92)	0.632
Processing Speed	101/98	9.86 (1.79)	10.02 (2.13)	-0.17 (-5.65, 5.32)	0.650
General Ability Index	101/98	3.99 (1.39)	3.21 (1.36)	0.78 (-3.06, 4.61)	0.799
Cognitive Proficiency Index	101/98	7.47 (1.25)	7.02 (1.26)	0.45 (-3.05, 3.95)	0.802
PSQ subscales (Behavioral)					
PSQ total score	121/107	-3.02 (1.34)	-3.73 (1.35)	0.71 (-3.04, 4.46)	0.515
Inattention	121/107	-0.28 (0.13)	-0.42 (0.12)	0.14 (-0.21, 0.49)	0.147
Hyperactivity/Impulsivity	121/107	-0.49 (0.17)	-0.21 (0.16)	-0.28 (-0.75, 0.18)	0.414
Behavioral and Social Problems	121/107	-0.59 (0.39)	-0.65 (0.33)	0.07 (-0.94, 1.07)	0.767
Emotional Problems	121/107	-0.59 (0.23)	-0.85 (0.27)	0.26 (-0.44, 0.96)	0.308
Somatic Symptoms	121/107	-0.41 (0.15)	-0.46 (0.18)	0.04 (-0.42, 0.51)	0.844
POMS subscales (Mood)					
Total Mood Disturbance	119/107	-0.15 (2.11)	-1.09 (1.77)	0.94 (-4.49, 6.37)	0.947
Tension	119/107	-0.33 (0.43)	-0.05 (0.39)	-0.28 (-1.42, 0.86)	0.485
Anger	119/107	0.22 (0.57)	-0.36 (0.51)	0.57 (-0.94, 2.09)	0.813
Fatigue	119/107	-0.39 (0.40)	0.00 (0.39)	-0.39 (-1.50, 0.71)	0.373
Depression	119/107	-0.01 (0.44)	-0.24 (0.38)	0.23 (-0.91, 1.38)	0.851
Vigor	119/107	0.17 (0.57)	-0.08 (0.63)	0.25 (-1.41, 1.91)	0.836
Confusion	119/107	0.08 (0.35)	-0.58 (0.30)	0.66 (-0.26, 1.57)	0.316
Esteem-Related Affect	119/107	-0.45 (0.43)	-0.05 (0.41)	-0.41 (-1.57, 0.76)	0.575

Note: The PP population included participants who adhered to the assigned intervention and completed post-intervention assessments according to the prespecified protocol. No imputation was performed for PP analyses. Values are presented as mean changes (SD) from baseline to post-intervention for each group. The Mean difference column represents the between-group difference in mean change (intervention minus control). All *p* values are derived from adjusted models controlling for baseline age, sex, BMI, and household income. The n_I/n_c indicate the number of participants included in the PP analysis for the intervention and control groups, respectively. Denominators vary across outcomes due to missing data. PP, per-protocol; WISC-IV, Chinese Wechsler Intelligence Scale for Children- Fourth Edition; POMS, Profile of Mood States- Brief; PSQ, Parent Conners Symptom Questionnaire; SD, standard deviation; BMI, body mass index.

Supplementary Table S12. Comparison of daily nutrient intakes between the intervention and control groups before the intervention

Nutrient	Intervention group	Control group	*P* value
Energy (kcal)	1429.54 (413.47)	1452.40 (368.24)	0.661
Protein (g)	72.03 (25.05)	71.91 (22.61)	0.971
Fat (g)	47.65 (24.63)	44.40 (15.47)	0.233
Carbohydrates (g)	191.79 (60.76)	201.03 (57.50)	0.242
Dietary Fiber (g)	15.81 (7.88)	15.96 (7.81)	0.888
Cholesterol (mg)	428.23 (243.72)	403.40 (224.85)	0.426
Ash (g)	10.07 (4.07)	10.38 (4.13)	0.575
Vitamin A (μg)	426.32 (288.58)	395.87 (215.26)	0.368
Carotene (mg)	1.91 (1.96)	1.71 (1.39)	0.390
Retinol (μg)	249.03 (225.72)	230.69 (168.01)	0.487
Thiamin (mg)	0.73 (0.35)	0.66 (0.23)	0.092
Riboflavin (mg)	0.89 (0.45)	1.00 (0.54)	0.095
Niacin (mg)	16.33 (7.16)	16.37 (6.76)	0.971
Vitamin C (mg)	111.97 (186.27)	91.24 (93.65)	0.287
Total Vitamin E (mg)	10.00 (5.76)	9.37 (5.51)	0.406
α-Vitamin E (mg)	4.09 (2.75)	4.10 (2.45)	0.987
(β+γ)-Vitamin E (mg)	3.49 (2.80)	3.04 (2.84)	0.236
δ-Vitamin E (mg)	1.20 (1.13)	1.09 (1.31)	0.497
Calcium (mg)	474.66 (201.93)	488.11 (183.11)	0.600
Phosphorus (mg)	947.22 (299.92)	958.48 (279.36)	0.770
Potassium (mg)	1895.17 (711.61)	1939.74 (797.90)	0.659
Sodium (mg)	1143.79 (594.93)	1195.83 (575.27)	0.505
Magnesium (mg)	231.56 (89.59)	238.58 (101.44)	0.583
Iron (mg)	15.19 (7.49)	16.97 (11.67)	0.178
Zinc (mg)	9.57 (4.10)	9.75 (3.70)	0.734
Selenium (μg)	36.83 (17.94)	34.43 (12.71)	0.244
Copper (mg)	1.34 (0.91)	1.19 (0.58)	0.132
Manganese (mg)	3.01 (2.45)	2.77 (1.52)	0.364

Note: Values are presented as mean (SD). *P* values were calculated using independent samples t-tests for between-group comparisons before the intervention. SD, standard deviation.
